# The role of private education in the selection of primary care careers in low and middle-income countries. Findings from a representative survey of medical residents in Brazil

**DOI:** 10.1186/s12960-020-0456-3

**Published:** 2020-02-17

**Authors:** Giuliano Russo, Alex J. Flores Cassenote, Aline G. Alves Guilloux, Mário César Scheffer

**Affiliations:** 1 Institute of Population Health Sciences, Queen Mary University of London, 58 Turner street, E1 2AB London, United Kingdom; 2 Departamento da Medicina Preventiva, Faculdade de Medicina da Universidade de São Paulo, CEP:01246-903, Av. Dr Arnaldo, 455, São Paulo, Brazil

**Keywords:** Primary care and UHC, Medical education in LMICs, Private medical schools, Physicians in Brazil, Choice of medical specialties, Family medicine in LMICs

## Abstract

**Background:**

Primary health care (PHC) doctors’ numbers are dwindling in high- as well as low-income countries, which is feared to hamper the achievement of Universal Health Coverage goals. As a large proportion of doctors are privately educated and private medical schools are becoming increasingly common in middle-income settings, there is a debate on whether private education represents a suitable mean to increase the supply of PHC physicians. We analyse the intentions to practice of medical residents in Brazil to understand whether these differ for public and private schools.

**Methods:**

Drawing from the literature on the selection of medical specialties, we constructed a model for the determinants of medical students’ intentions to practice in PHC, and used secondary data from a nationally representative sample of 4601 medical residents in Brazil to populate it. Multivariate analysis and multilevel cluster models were employed to explore the association between perspective physicians’ choice of practice and types of schools attended, socio-economic characteristics, and their values and opinions on the profession.

**Results:**

Only 3.7% of residents in our sample declared an intention to practice in PHC, with no significant association with the public or private nature of the medical schools attended. Instead, having attended a state secondary school (*p* = 0.028), having trained outside Brazil’s wealthy South East (*p* < 0.001), not coming from an affluent family (*p* = 0.037), and not having a high valuation of career development opportunities (*p* < 0.001) were predictors of willingness to practice in PHC. A low consideration for quality of life, for opportunities for treating patients, and for the liberal aspects of the profession were also associated with future physicians’ intentions to work in primary care (all *p* < 0.001).

**Conclusions:**

In Brazil, training in public or private medical schools does not influence the intention to practice in PHC. But students from affluent backgrounds, with private secondary education, and graduating in the rich South East were found to be overrepresented in both types of training institutions, and this is what appears to negatively impact the selection of PHC careers. With a view to increasing the supply of PHC practitioners in middle-income countries, policies should focus on opening medical schools in rural areas and improving access for students from disadvantaged backgrounds.

**Supplementary information:**

**Supplementary information** accompanies this paper at 10.1186/s12960-020-0456-3.

## Background

Primary health care (PHC) services are essential to improve the health of populations throughout the world. The 1979 Alma Ata declaration placed PHC at the core of those health policies aimed at reducing mortality and morbidity, as well as at maximising the impact of health spending in low- and middle-income countries (LMIC) [1]. However, shortages of primary care physicians are increasingly reported throughout the world, as fewer medical students select PHC and family medicine specialties in rich [2–4] as well as poorer countries [5]. As primary care specialties typically fetch comparatively lower salaries than hospital ones [6], and rank consistently low for professional prestige in the medical community [7], scholars and policy-makers worry that filling general practice positions in public healthcare systems will become increasingly difficult, particularly for low-income and rural areas [8].

Besides economic motifs, multiple drivers are believed to be at the heart of medical students’ choice of specialties. Evidence exists linking preference for specific specialties to medical students’ socio-economic characteristics [9], to their valuation of future job opportunities [10], and to the characteristics of the training received [11]. Labour market forces and prospective revenues have also been identified as major influencing factors in the selection of specialties, with the expected Rate of Return to Education for specific medical professions being a key, and still largely unexplored, determinant of students’ decisions [12].

Some conceptual frameworks of medical education [11] also consider the public or private nature of medical schools as an influence on students’ choice of specialty, and evidence from the USA shows that elite, private schools do a poor job in producing future primary care physicians [2] [13]. As privately run medical schools are becoming increasingly common worldwide, a debate exists on whether using private schools to boost the supply of physicians would be a suitable option for HICs and LMICs [14]. Overall, the evidence from low- and middle-income countries is scant and mostly descriptive [15–17]; a systematic review on the selection of primary care specialties in LMICs [18] identified drivers specific to lower-income settings, like students’ understanding of rural needs, and the intellectual challenge of supporting a country’s development.

Brazil’s advances towards universal coverage have been significant but marked by substantial inequalities, despite the creation in 1988 of a publicly funded, free at the point of use Unified Healthcare System (SUS) [19]. Although Brazil’s primary care Family Health Programme is credited to have contributed to the country’s recent health gains [20], the barriers for medical students to work in primary care settings have long been highlighted [21], and poor motivation is widely thought to be at the root of students’ weak demand for primary care training [22]. In order to fill the existing vacancies in primary care and rural areas foreign GPs were recruited though the More Physician programme [23], and a substantial number of new private medical schools have been authorised to increase the supply of graduates [24].

At the time of writing (December 2019), there were 341 medical schools in Brazil, 222 of which privately funded, offering overall 35 542 places per year. Training is free at public institutions, but places are few and competition steep, with selection based on a national threshold for secondary school average grades (*Enem*), and institution-specific entry exams (*Vestibular*) [25]. Tuition fees at private universities average approximately USD25,000 per year. Places are limited in private institutions too, but competition is less intense, as lower thresholds are set for Enem and Vestibular exams scores [26]. Critics have voiced concerns on the ability of these new, private medical schools to produce the PHC doctors that the Brazilian health system needs [27, 28].

## Methods

### The methodological approach

This study sets out to identify any systematic difference in the intentions to practice in primary care settings in a representative sample of newly qualified physicians from public and private medical schools in Brazil. We use multivariate multilevel statistical analysis to explore the association between the selection of PHC specialties and a range of possible determinants of choice, identified in the medical education literature. We adapted Bland, Meurer, and Maldonado’s 1995 model of the determinants of students’ choices of specialties [11] and divided potential influencing factors into the following: (a) student’s personal and socio-economic characteristics (gender, family income, age); (b) type of school attended (secondary school, medical school of training, urban or rural location of the schools); (c) student’s needs to satisfy personal and societal expectations (intrinsic and extrinsic motivation); (d) perception of specialty characteristics (prestige, working hours, contact with patients, social role and prospective earnings. We identified suitable variables from the ‘Profile and perceptions of new graduates in medicine in Brazil’ survey study [29] as proxies for the above factors.

Our hypothesis was that proxy variables for gender [30], family background [31], and socially sensitive views on the profession [32, 33] were going to show a positive association with the intention to practice in primary care settings. As per the influence of medical schools, we assumed that students trained in public institution were going to display a greater inclination to select primary care practice [2, 13].

### Data set and variables

We used secondary data from a representative cross-sectional survey conducted in 2015 by the São Paulo University [29], to perform descriptive, multivariate logistic, and multilevel analysis on the effect of a range of variables on students’ declared intention to practice in primary care settings. The survey dataset had 4601 respondents from a population of 16 323 eligible newly graduated physicians.^1^ The original survey was distributed electronically to all the medical school graduates previously registered with one of Brazil’s 27 Regional Medical Councils in 2015. To overcome the possible response bias from the students accepting to participate in the survey, the sample was stratified according to sex, public or private nature of the medical school, and geographic origin, as identified by the medical school attended. Weighting was used to ensure the representativeness of those variables in the same proportion as they were observed in the population. The survey was approved by the University of São Paulo Medical School’s Research Ethics Committee. The details on the survey sampling and treatment of the original variables have been published elsewhere [34].

The survey questionnaire included 104 multiple choice questions organised in 10 sections covering medical students’ socio-economic characteristics, assessment of the medical training received, valuation of aspects of the medical profession, future choice of specialty, and professional expectations (see the survey instrument in the background files). Students’ intention to practice in primary care settings was the main outcome variable in our study. We provide below a description of the relevant variables used in our analysis (Table 1).

**Table 1:** Questionnaire variables used in the analysis

Type of variable	Description
Outcome variable: intention to practice in PHC settings	In what type of health care institutions would you exclusively like to work? (0) Basic health unit or Family Medicine Programme; (1) Hospital, clinic, private office, clinical lab, pharmaceutical industry, university or other.
Sex	How would you define your gender? (0) Female (1) Male (2) Other/prefer not to answer
Geographical location of medical school attended	In what region of the country was the medical school you attended? (0) North (1) North East (2) South (3) South East (4) Centre-West
Type of medical school	What type of medical school did you attend? (0) Private (1) Public
Family income	What is your household income? (0) Below 10 times the national minimum salary (1) Above 10 times the minimum salary
Parents’ education level	Did one of your parents hold a tertiary education title? (0) Yes (1) No
Type of secondary school attended	What type of secondary school(s) did you mostly attend? (0) All or mostly public schools (1) All or mostly private schools
Factors persuading to stay in the job (a)	Your employment offers a career development plan (0) Yes (1) No
Factors persuading to stay in the job (b)	Your employment offers quality of life (0) Yes (1) No
Student’s motivation for choosing a specific specialty (a)	You prefer working in the public sector (0) Yes (1) No
Student’s motivation for choosing a specific specialty (b)	Your specialty offers the opportunity to interact with people (0) Yes (1) No
Student’s motivation for choosing a specific specialty (c)	Your selected specialty offers the opportunity to treat people and solve health problems (0) Yes (1) No
Characteristics of the specialty selected (a)	The profession’s liberal and independence characteristics (0) Yes (1) No
Characteristics of the specialty selected (b)	The social responsibility of the medical profession (0) Yes (1) No

### Data analysis

We performed descriptive, bivariate, and multilevel clustered analysis on the dataset described above. As the students’ response rate differed across strata in our sample, we investigated cluster effects in the outcome variable, and a high variability was detected between clusters (see the cluster distribution in supplementary material file 1), which was also visible in the difference of frequencies for variables in the weighted and unweighted sample (Table 2). This justified the use of Generalised Linear Mixed Models (GLMMs) as an alternative to conventional models [35]. GLMMs extend standard generalised linear methods by allowing for random or cluster-specific effects in the linear predictor; the inclusion of random effects in the linear predictor reflects the idea that there is natural heterogeneity across clusters in their regression coefficients. Such method has been used before in health services research [36].

**Table 2:** Characteristics of the graduates’ population that responded YES for the outcome variable ‘I would like to work exclusively in PHC’, by weighted and unweighted frequencies (confidence interval 95%)

Variables	*N*	Unweighted (sample)			Weighted (population)			Total sample
		%	CI95%		%	CI95%		
			Lower	Upper		Lower	Upper	
Sex								
Female	97	59.5%	51.9%	66.8%	66.6%	28.1%	91.0%	1 880
Male	66	40.5%	33.2%	48.1%	33.4%	9.0%	71.9%	1 570
Trained in a medical school in the South East region								
Yes	50	30.7%	24.0%	38.0%	61.7%	25.1%	88.6%	1 665
No	113	69.3%	62.0%	76.0%	38.3%	11.4%	74.9%	1 785
Type of medical school								
Private	92	56.4%	48.8%	63.9%	71.7%	36.8%	91.6%	1 909
Public	71	43.6%	36.1%	51.2%	28.3%	8.4%	63.2%	1 541
Family income > 10 minimum salary								
Yes	68	42.8%	35.3%	50.5%	43.8%	37.2%	50.6%	1 898
No	91	57.2%	49.5%	64.7%	56.2%	49.4%	62.8%	1 476
Father or mother with tertiary education								
Yes	110	67.5%	60.0%	74.3%	69.1%	65.3%	72.7%	2 723
No	53	32.5%	25.7%	40.0%	30.9%	27.3%	34.7%	726
Type of secondary school attended								
Mostly in public school(s)	48	29.8%	23.2%	37.2%	27.7%	21.1%	35.5%	584
Mostly in private school(s)	113	70.2%	62.8%	76.8%	72.3%	64.5%	78.9%	2 830
Factors for deciding to remain in the job								
Having a career development plan								
Yes	39	24.5%	18.3%	31.6%	24.2%	18.3%	31.3%	1 594
No	120	75.5%	68.4%	81.7%	75.8%	68.7%	81.7%	1 799
Quality of life								
Yes	71	44.7%	37.1%	52.4%	39.0%	30.0%	48.9%	2 333
No	88	55.3%	47.6%	62.9%	61.0%	51.1%	70.0%	1 060
Incentivising factors								
Preference for working in the public sector								
Yes	100	61.7%	54.1%	69.0%	67.2%	58.9%	74.6%	1 729
No	62	38.3%	31.0%	45.9%	32.8%	25.4%	41.1%	1 697
Interaction with people								
Yes	112	71.8%	64.4%	78.4%	72.0%	66.3%	77.0%	2 403
No	44	28.2%	21.6%	35.6%	28.0%	23.0%	33.7%	944
Opportunity for treating diseases and resolving health problems								
Yes	92	59.0%	51.1%	66.5%	58.2%	45.1%	70.3%	2 548
No	64	41.0%	33.5%	48.9%	41.8%	29.7%	54.9%	799
The profession’s liberal and independence characteristics								
Yes	28	17.9%	12.5%	24.5%	17.4%	12.7%	23.3%	1 076
No	128	82.1%	75.5%	87.5%	82.6%	76.7%	87.3%	2 271
Physician’s social responsibility								
Yes	58	37.2%	29.9%	44.9%	43.9%	33.6%	54.8%	1 175
No	98	62.8%	55.1%	70.1%	56.1%	45.2%	66.4%	2 172

Bivariate analysis was performed considering cluster effects to examine the association between the selected outcome variable: ‘I would like to work exclusively in primary care settings’ and the selected explanatory variables. A crude odd ratio (OR) from this evaluation was first applied to assess the impact of individual factors on outcomes [37]. The generalised linear mixed model with a binomial distribution was used to estimate the adjusted odds ratio (ORa). The ‘enter’ method was used for the variables’ selection, and ANOVA tests were used to verify the equality hypothesis among the different models. Data were shown as absolute frequency and proportion with a 95% confidence interval.

As the cluster of students trained in medical schools from the South East was dominant in our sample, we constructed two GLMM models, the second of which excluded the geographical location of medical school variable to test for the significance of obscured variables in other clusters. The adjustment of different models was verified by indicators of residual deviance and the Akaike information criterion (AIC) [38].

The database was exported to the Statistical Package for the Social Sciences (SPSS) version 26 for Windows (International Business Machines Corp, New York, USA) and R-GUI version 3.5.3 [39] for statistical treatments. All the significance levels were set to *p* < 0.05.

## Results

Female students were the majority (62.1%) in our graduates’ population. Almost three quarters (76.8%) of all the students in our sample were enrolled in a medical school in the South East of the country. The majority of students in our sample were enrolled in private medical schools (74.2%), from families with a household income greater than 10 times the minimum wage (58.1%), with one of the parents holding a higher education degree (80.5%), having predominantly studied in private secondary schools (85.6%).

**Fig. 1:**
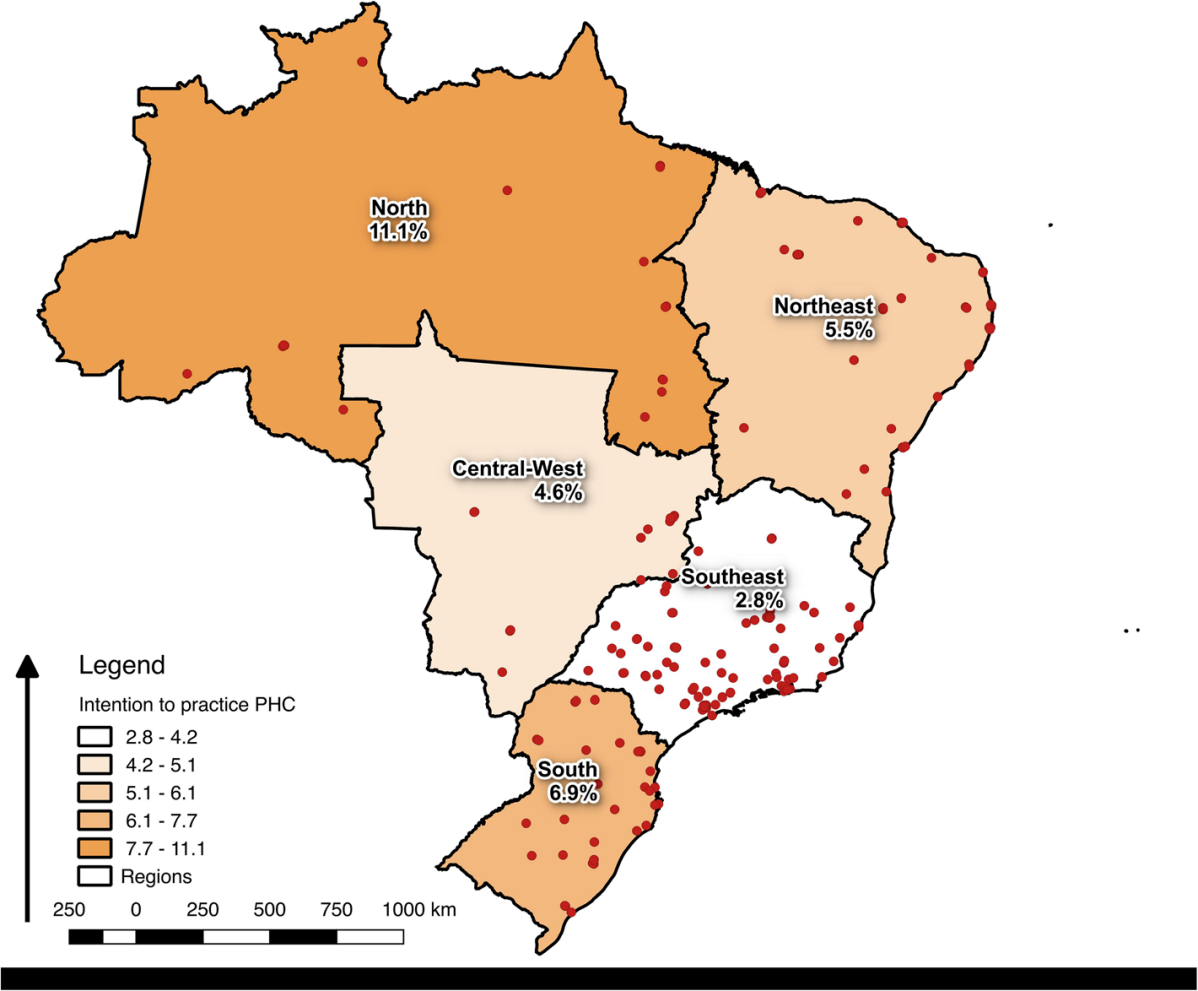
Medical schools per Brazil’s region and proportion of residents declaring their intentions to practice in PHC

We obtained 3450 valid responses in our sample. Across the country’s medical schools included in the 2015 survey (203), only 3.7% (163) of the medical residents declared their preference to work exclusively in primary care institutions, with substantial differences across rural and urban areas (Fig. 1). The highest proportions of residents planning to practice in primary care were recorded in the North of Brazil (11.1%), while the lowest was recorded for the wealthy and populous South East (2.8%).

Opportunities of interaction (73.6%) and for treating people (78.4%) were considered as the most appealing characteristics of the medical profession. Conversely, independence of the profession (33.7%) and physicians’ social responsibilities were not signalled by many (35.6%) as a reason for selecting the profession and a specialty. The bivariate analysis showed a strong negative (OR = 2.180) association between the intention to practice in primary care and medical school’s South East geographic location (*p* = 0.001), household income (OR = 1.760, *p* = 0.001), parents’ higher education (OR = 1.870, with *p* < 0.001), and having attended a private secondary school (OR = 0.469 with *p* < 0.001), as well as with a range of socio-economic and expressed preferences variables (Table 3). The public nature of the medical school attended was not a significant factor in the graduates’ intention to practice in PHC.

**Table 3:** Results from the multilevel cluster analysis

	Outcome variable		Bivariate analysis			*p*	Model I			*p*	Model II			*p*
	*N*	% (IC95%)	OR	IC95%			ORa	IC95%			ORa	IC95%		
				Lower	Higher			Lower	Higher			Lower	Higher	
Sex														
Female	97	59.5 (51.9–66.8)	Reference				Reference				Reference			
Male	66	40.5 (33.2–48.1)	0.188	0.586	1.110	0.188	0.775	0.546	1.100	0.155	0.794	0.560	1.127	0.196
Trained in a medical school in the South East region														
Yes	50	30.7 (24–38)	Reference				Reference							
No	113	69.3 (62–76)	2.180	1.550	3.060	0.001	2.105	1.430	3.085	< 0.001				
Type of medical school														
Private	92	56.4 (48.8–63.9)	Reference				Reference				Reference			
Public	71	43.6 (36.1–51.2)	0.954	0.695	1.310	0.771	0.786	0.550	1.120	0.184	0.890	0.627	1.264	0.196
Family income > 10 minimum salary														
Yes	68	42.8 (35.3–50.5)	Reference				Reference				Reference			
No	91	57.2 (49.5–64.7)	1.760	1.280	2.440	0.001	1.148	1.025	2.163	0.037	1.489	1.023	2.167	0.038
Father or mother with tertiary education														
Yes	110	67.5 (60–74.3)	Reference				Reference				Reference			
No	53	32.5 (25.7–40)	1.870	1.330	2.620	< 0.001	1.126	0.835	1.903	0.270	1.332	0.088	2.013	0.175
Type of secondary school attended														
Mostly in public school(s)	48	29.8 (23.2–37.2)	Reference				Reference				Reference			
Mostly in private school(s)	113	70.2 (62.8–76.8)	0.469	0.320	0.650	< 0.001	0.629	0.416	0.951	0.028	0.630	0.417	0.951	0.028
Factors for deciding to remain in the job														
Having a career development plan														
Yes	39	24.5 (18.3–31.6)	Reference				Reference				Reference			
No	120	75.5 (68.4–81.7)	0.351	0.245	0.507	< 0.001	0.456	0.310	0.671	< 0.001	0.458	0.612	0.674	< 0.001
Quality of life (opportunity to earn a high income)														
Yes	71	44.7 (37.1–52.4)	Reference				Reference				Reference			
No	88	55.3 (47.6–62.9)	2.880	2.090	3.970	< 0.001	2.136	1.556	2.999	< 0.001	2.063	1.470	2.890	< 0.001
Incentivising factors														
Preference for working in the public sector														
Yes	100	61.7 (54.1–69)	Reference	–	–		Reference				Reference			
No	62	38.3 (31–45.9)	0.618	0.447	0.854	0.004	0.721	0.500	1.038	0.078	0.717	0.498	1.032	0.073
Interaction with people														
Yes	112	71.8 (64.4–78.4)	Reference				Reference				Reference			
No	44	28.2 (21.6–35.6)	1.000	0.700	1.429	0.999	1.151	0.782	1.169	0.475	1.109	0.756	1.628	0.596
Opportunity for treating diseases and resolving health problems														
Yes	92	59 (51.1–66.5)	Reference				Reference				Reference			
No	64	41 (33.5–48.9)	2.320	1.670	3.230	< 0.001	1.853	1.299	2.631	< 0.001	1.934	1.361	2.748	< 0.001
The profession’s liberal and independence characteristics														
Yes	28	17.9 (12.5–24.5)	Reference				Reference				Reference			
No	128	82.1 (75.5–87.5)	2.230	1.470	3.380	< 0.001	1.613	1.016	2.561	0.043	1.771	1.121	2.802	0.014
Physician’s social responsibility														
Yes	58	37.2 (29.9–44.9)	Reference				Reference				Reference			
No	98	62.8 (55.1–70.1)	0.910	0.652	1.260	0.579	0.806	0.563	1.153	0.238	0.797	0.559	1.137	0.211

When conducting the multivariate multilevel analysis in our Model I, being enrolled in a medical school outside the South East, still presented a strong positive association with the intention to practice in primary care (*p* < 0.001). Not coming from a wealthy family (*p* = 0.037), and not having studied in a private secondary school (*p* = 0.028), were the characteristics with a positive, significant association with the PHC-dependent variable. As for students’ stated opinions and valued characteristics of the profession, ‘Seeking a defined career development path’, ‘Seeking quality of life’ in the medical profession, and ‘Valuing the opportunity to treat patients’ as key features of the profession all had negative association with the expressed preference for PHC practice (all with *p* < 0.001).

Somewhat unexpectedly, gender, the public/private nature of medical schools, and having at least one parent with a higher education degree were not statistically significant in Model I of our multilevel analysis (*p* = 0.155, *p* = 0.184 and *p* = 0.270, respectively). Also valuing interaction with people/patients, and the social responsibility of the medical profession, was not significantly associated with PHC practice.

Because of the dominance of residents enrolled in medical schools in the populous São Paulo area, in Model II we excluded the ‘Enrolment in a medical school in the South East’ variable to explore associations outside such region. The same variables as Model I resulted significant also in Model II, with the addition of ‘Valuing the independence and liberal aspect of the profession’, which was negatively associated with the intention to practice in PHC settings (*p* = 0.014). Model I (including the South East geographical location variable for medical schools attended) presented a slightly better Adjusted Akaike and Bayesian criterion values than Model II, which is suggestive of its superior ability to explain the variance in the sample.

## Discussion

Our analysis of a representative sample of newly qualified medical students in Brazil showed that the public or private nature of the medical school attended was not associated with students’ intentions to practice in PHC settings. Instead, having trained in a medical school outside the South East, not coming from an affluent family, not having attended a private secondary school, and not having a high valuation for career development plans were significant predictors of graduates’ willingness to practice in PHC. Unexpectedly, a low valuation of quality of life, of the opportunities offered for treating patients, and for the independent and liberal aspects of the medical professions were associated with students’ intention to work in primary care settings.

These conclusions are affected by specific data limitations, as ours was a cross-sectional study of perspective physicians’ intentions to practice, and it was not possible to verify whether these truly ended up working in the specialties indicated. However, despite the accepted limitations of intentions data [40], there seems to be a consensus that stated intentions are a valid predictor of planned behaviour [41] and have been used extensively to study medical students’ choice of specialties [7, 10, 42]. Most of our survey variables were also qualitative and categorical, which reduced the scope for statistical analysis. The low number of students declaring their intention to practice in PHC (163 out of 3 450) may have also affected the internal validity of our analysis. We also acknowledge the limitation of our adopted output variable, as a different formulation of the question posed (‘In what type of health care institutions would you exclusively like to work?’) may have produced different responses from medical residents.

The finding that the public/private nature of medical schools does not influence the intention to practice in PHC is somewhat counterintuitive and appears to contradict the expectations of Brazilian [27, 28] and international scholars [13]. A possible explanation for this may be found in the overrepresentation of students from affluent backgrounds in public medical schools, and in the demanding admission tests, granting places only to the highest performing students, typically schooled privately [43]. This would be consistent with an argument made by studies from other countries that the type of secondary schools attended by medical students would be a mediating factor between public nature of medical school, performance, and selection of specialties [44].

Having trained in a medical school in Brazil’s South East was found to negatively affect students’ inclination to engage with primary health care (Fig. 1). The buoyant, commercial, and hospital-oriented nature of the physician labour market in the wealthy São Paulo region [45] may offer an explanation for this result, as recent evidence shows that more physicians engage in dual practice in that state than anywhere else in Brazil [46]. Medical students trained in São Paulo would be therefore be exposed since inception to a professional culture oriented towards hospital specialties and practice in the private sector, something that would shape their professional expectations and selection of future practice. If confirmed, this finding lends support to those theories suggesting that the teaching culture of the medical school attended [11], but also the opportunities for employment offered by the local labour market [12], are important drivers of student’s choice of specialty.

Counterintuitively, we found quality of life and opportunities for treating people to be negatively associated with PHC practice, which somewhat contradicts previous studies on PHC students’ motivation [11, 47]. While it is possible that security and remoteness of PHC facilities remain a concern for health workers’ in Brazil [48], it is likely that our survey respondents interpreted the question on quality of life as ‘opportunities to earn a high (er) income’, which is an idiosyncratic translation in Brazilian Portuguese for ‘quality of life’. Therefore, this finding should be interpreted as profit-minded students not planning to engage in PHC jobs, which would be entirely consistent with the international evidence on this regard [49]. As for the opportunity to treat people, consideration needs to be given to the fact that in Brazil’s own conception of medicine, treating and healing is associated more with curative, hospital-based services, whereas primary health care is linked more to preventive, community-based services [50].

Despite the specificities of Brazil’s education system and health labour market, our findings carry implications for medical education policies in other low- and middle-income countries such as India, where private medical schools stand accused of offering additional avenues into the profession for the wealthy [51]. As for the UK case [43, 44], our findings suggest that, precisely because of their demanding academic requirements, state medical schools may already have been captured by elite students from fee-paying secondary schools. The implications of this being that, rather than just selecting candidates on academic merit, medical schools should grant admission to PHC-minded students from disadvantaged background, if the future supply of primary care physicians is to be increased. Likewise, training physicians in institutions located in rich and business-oriented areas may not help the cause of primary healthcare in middle-income countries. New medical schools should be located closer to the regional market health planners’ want to supply [12], with a view to attracting students from and with similar values of the communities that need to be served.

## Conclusions

There is an ongoing debate on the influence of the public or private nature of secondary and medical schools on the selection of PHC specialties in high-, low-, and middle-income countries. We used a model of determinants of specialty choice and survey data from Brazil to explore the association between intention to practice in PHC and personal characteristics, schools attended, socio-economic background, values, and opinions. Our multilevel cluster analysis revealed that geographical location of the medical school, having attended a public secondary school, coming from a lower-income household, and holding a non-profit-oriented outlook of the medical profession, are significant factors influencing the selection of PHC specialties in Brazil. Conversely, gender and the public/private nature of the medical schools attended did not display a significant association with intention to practice in primary care.

Our study carries implications for policies aimed at strengthening PHC training worldwide, as it appears to show that selection of suitable students’ profiles, and medical schools’ geographical location, are more relevant factors than the public or private nature of training institutions for the development of a national PHC workforce. A more balanced proportion of students from disadvantaged backgrounds should be sought in national secondary education and medical schools, as these individuals appear to hold values and aspirations more conducive to future practice in PHC settings.

## Authors’ conflict of interest statement

None declared

## Supplementary information

Additional file 1. Cluster distribution.

Additional file 2. Survey questionnaire.

## Footnotes

1 Unlike other countries, in Brazil physicians are granted autonomy and licence to work before specialisation upon completion of their 6-year medicine degree.
