# Supplementary material for: The role of private education in the selection of primary care careers in low and middle-income countries. Findings from a representative survey of medical residents in Brazil
Source: Hum Resour Health. 2020 Feb 17;18:11. doi: 10.1186/s12960-020-0456-3 (PMC7027019; doi:10.1186/s12960-020-0456-3)
Supplement: Supplementary file 1 — Cluster distribution. [file 12960_2020_456_MOESM1_ESM.docx]

**Supplementary material file 1: Cluster distribution**

| **Cluster** | **Description** | **Total** | **SPP** | **%** | **IC95%** | |
| --- | --- | --- | --- | --- | --- | --- |
|  |  |  |  |  | **Inferior** | **Superior** |
| CLUSTER 1 | Female - Public - Centre-West | 35 | 1 | 0.6% | 0.1% | 2.8% |
| CLUSTER 2 | Female - Public - North-East | 178 | 6 | 3.7% | 1.6% | 7.4% |
| CLUSTER 3 | Female - Public - North | 101 | 8 | 4.9% | 2.3% | 9.0% |
| CLUSTER 4 | Female - Public - South-East | 317 | 10 | 6.1% | 3.2% | 10.6% |
| CLUSTER 5 | Female - Public - South | 151 | 7 | 4.3% | 1.9% | 8.2% |
| CLUSTER 6 | Female - Private - Centre-West | 45 | 4 | 2.5% | 0.8% | 5.7% |
| CLUSTER 7 | Female - Private - North-East | 190 | 16 | 9.8% | 6.0% | 15.1% |
| CLUSTER 8 | Female - Private - North | 40 | 7 | 4.3% | 1.9% | 8.2% |
| CLUSTER 9 | Female - Private - South-east | 620 | 20 | 12.3% | 7.9% | 18.0% |
| CLUSTER 10 | Female - Private - South | 203 | 18 | 11.0% | 6.9% | 16.5% |
| CLUSTER 11 | Male - Public - Centre-West | 45 | 1 | 0.6% | 0.1% | 2.8% |
| CLUSTER 12 | Male - Public - North-East | 198 | 10 | 6.1% | 3.2% | 10.6% |
| CLUSTER 13 | Male - Public - North | 101 | 11 | 6.7% | 3.6% | 11.4% |
| CLUSTER 14 | Male - Public - South-east | 299 | 9 | 5.5% | 2.8% | 9.8% |
| CLUSTER 15 | Male - Public - South | 116 | 8 | 4.9% | 2.3% | 9.0% |
| CLUSTER 16 | Male - Private - Centre-West | 34 | 4 | 2.5% | 0.8% | 5.7% |
| CLUSTER 17 | Male - Private - North-East | 140 | 6 | 3.7% | 1.6% | 7.4% |
| CLUSTER 18 | Male - Private - North | 51 | 4 | 2.5% | 0.8% | 5.7% |
| CLUSTER 19 | Male - Private - South-east | 429 | 11 | 6.7% | 3.6% | 11.4% |
| CLUSTER 20 | Male - Private - South | 157 | 2 | 1.2% | 0.3% | 3.9% |
| Total |  | 3450 | 163 | 4.7% | 4.1% | 5.50% |
